# Supplementary material for: Metabolic Profile of Scytalidium parasiticum-Ganoderma boninense Co-Cultures Revealed the Alkaloids, Flavonoids and Fatty Acids that Contribute to Anti-Ganoderma Activity
Source: Molecules. 2020 Dec 16;25(24):5965. doi: 10.3390/molecules25245965 (PMC7767070; doi:10.3390/molecules25245965)
Supplement: Supplementary file 1 [file molecules-25-05965-s001.pdf]

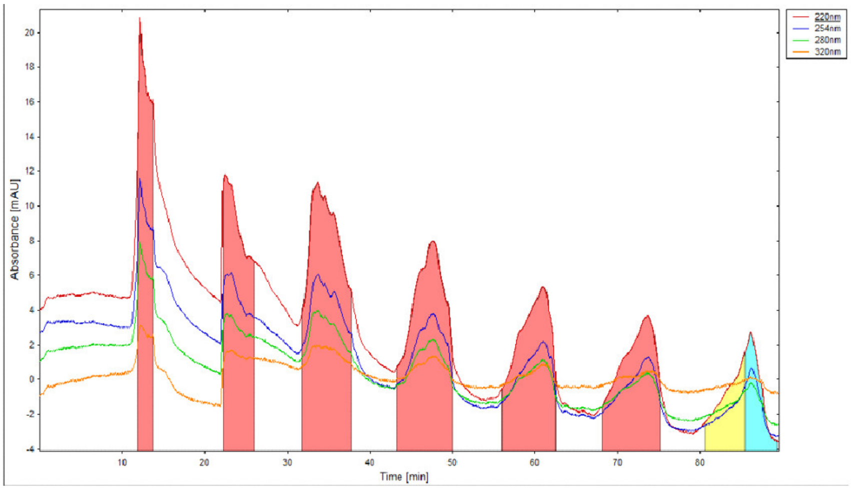

Figure S1. Chromatogram crude extract of *G.boninense-S.parasiticum* co-culture in recycling preparative HPLC. Highlighted in red is the fraction that has been collected for antifungal test.
